# Predictive equations for dietary energy are improved when independently developed for dry and wet food which could benefit both the pet and the environment

**DOI:** 10.3389/fvets.2023.1104695

**Published:** 2023-02-21

**Authors:** Dennis E. Jewell, Matthew I. Jackson

**Affiliations:** ^1^Department of Grain Science and Industry, Kansas State University, Manhattan, KS, United States; ^2^Hill's Pet Nutrition, Topeka, KS, United States

**Keywords:** dogs (*Canis lupus familiaris*), cats (*Felis catus*), energy predication, sustainability, environmental impact

## Abstract

**Introduction:**

Measuring energy availability through metabolizable energy feeding studies is the “gold standard” for establishing metabolizable energy concentration. However, predictive equations are often used to estimate metabolizable energy in dog and cat pet foods. The goal of this work was to evaluate the prediction of energy density and compare those predictions to each other and the energy needs of the individual pets.

**Methods:**

Feeding studies used 397 adult dogs and 527 adult cats on 1,028 canine and 847 feline foods. Individual pet results for the estimate of metabolizable energy density were used as outcome variables. Prediction equations were generated from the new data and compared to previously published equations.

**Results and discussion:**

On average the dogs consumed 747 kilocalories (kcals) per day (SD = 198.7) while cats consumed 234 kcals per day (SD = 53.6). The difference between the average prediction of energy density and the measured metabolizable energy varied from the modified Atwater prediction 4.5%, 3.4% (NRC equations), 1.2% (Hall equations) to the new equations calculated from these data at 0.5%. The average absolute values of the differences between measured and predicted estimates in pet foods (dry and canned, dog and cat) are: 6.7% (modified Atwater), 5.1% (NRC equations), 3.5% (Hall equations) and 3.2% (new equations). All of these estimates resulted in significantly less variation in the estimate of the food expected to be consumed than the observed variation associated with actual pet consumption to maintain body weight. When expressed as a ratio of energy consumed to metabolic body weight (weight in kilograms^3/4^) the within species variation in energy consumed to maintain weight was still high as compared to the energy density estimates variance from measured metabolizable energy. The amount of food offered as the central point in a feeding guide, based on the prediction equations, would on average result in an average variance between 8.2% error in the worst case estimate (feline dry using modified Atwater estimates) and approximately 2.7% (the new equation for dry dog food). All predictions had relatively small differences in calculating food consumed when compared to the differences associated with the variation in normal energy demand.

## Introduction

The use of commercially available pet foods provides for the nutritional needs of dogs and cats. The foods designed for meeting the complete nutritional needs of pets make this claim through testing or product analysis which adequately shows they are sufficient to meet the required nutritional needs. In order to formulate these foods an estimate of energy density is used with an estimate of energy requirements. The estimate of the energy requirements and thus daily food intake offered is often based on an understanding of expected energy use associated with age and lifestyle (1). The estimates of energy density of the foods are then used to calculate the amount of food to be offered each day to each pet. Because energy density changes the amount of food consumed, the knowledge of energy density also drives the nutrient density of all other nutrients. The complete combustion of a pet food is used to measure the gross energy concentration (1) while metabolizable energy (ME) is either measured through a feeding study or by estimation through use of calculations based on measured concentrations of gross energy, moisture, protein, fat, ash and fiber. The standard for measuring ME is a feeding study where the energy lost in the feces and urine is subtracted from the gross energy of the food to calculate the available ME for use by the pet. The Association of American Feed Control Officials (AAFCO) (2) has suggested feeding protocols which when applied determine the energy density of dog and cat foods. The prediction equations for estimating energy density include the modified Atwater equations suggested by AAFCO (2) which are the result of modifications for dogs (3) and for cats (4). These factors do not change for the energy digestibility of the foods but have been shown to reasonably be used as estimates for commercial dog and cat foods from 85 to 75% energy digestibility (5). However, as many pet foods have energy digestibility above 85% there has been a number of other energy estimate equations suggested (6–8). The analytical basis used in these predictions requires endpoints with standard analysis that are highly repeatable. These equations have started with gross energy (GE) and crude fiber (9). The equations of the NRC (1) are such equations and are based on using the gross energy of the food as a starting point and then modifying that estimate by factors associated with dietary fiber (either crude fiber or total dietary fiber) as well as dietary protein concentration. The equations of Hall et al. (7) use gross energy and then modify that energy density by factors associated with dietary concentration of moisture, protein, fat, ash and crude fiber. Depending on the foodstuffs and foundationally the energy digestibility of the food different equations are best at predicting measured energy density (10). Because of limits on available data, the equations most used have been developed to estimate energy density in both dry and wet foods. As ingredients and energy digestibility are often different between dry and wet pet foods there is a reasonable possibility of improved equations tailored to each form. Because pet foods are often formulated and sold without measuring energy density through feeding studies they have energy estimates that may result in nutrients either being under formulated or over formulated as nutrient concentration is scaled to dietary energy density. The best practice of feeding pets is to adjust energy intake through changing food offering (which maintains an optimal body composition). If dietary energy density is overestimated than the initial feeding guide will underestimate food use, as food intake is adjusted up to maintain body weight there is a subsequent overconsumption of nutrients. Therefore, if energy density is not correctly known there can be an over intake of nutrients and subsequent increased excretion leading to an increased environmental footprint. There is therefore a constant desire to improve dietary energy prediction in order to optimize feeding guides, enhance the balance of nutrients to energy and improve the environmental footprint of pet food. This study uses 1,875 digestibility studies to evaluate the energy density predictions of the modified Atwater factors, the NRC (crude fiber) equations, the Hall equations, and generates new equations associated with a subset of these data. The data were separated into two groups with approximately 30% of the data randomly assigned to a group which was not used to generate the new prediction equations but reserved as a group to test the equations accuracy.

## Materials and methods

The studies reported here were completed over a period of 24 years. There were 397 healthy adult dogs and 527 healthy adult cats included in these studies (Table 1). There were 6,155 individual canine energy digestibility data points used and 4,828 feline individual results used. The digestibility studies were conducted by following AAFCO methodology where energy intake is measured through measuring food intake while non-metabolizable energy is measured through fecal energy output with urinary energy loss calculated through calculation of urinary nitrogen (2). In short, these studies consist of two phases, in the first phase there is a 7 day period to allow the dogs and cats to become acclimated to the food as well as to adjust food intake in order to maintain weight as needed. The second phase is the next 5 days (120 h) which is used for total fecal collection. During this second phase food offered is held constant (at the amount determined in phase one to maintain weight). The length of the two periods allows sufficient time for control of energy intake to maintain weight (period 1) and enough fecal collection so that timing of bowel movements does not excessively change estimates of digestibility (period 2). All pets had access to water at all times and daily food offerings of the amount which maintains body weight.

**Table 1:** Means (and standard deviations) of pet weight, age, and energy use.

	**Weight (kg)**	**95% Confidence interval**	**Age (years)**	**95% Confidence interval**	**Energy intake kcalories/day**	**95% Confidence interval**	**Energy to weight ratio^a^**	**95% Confidence interval**
Dogs^b^	11.5 (3.01)	11.3–11.7	6.2 (2.68)	6.1–6.3	747 (198.7)	737–757	120 (23.9)	119–121
Cats^c^	5.1 (1.22)	5.0–5.2	6.6 (2.67)	66.5–6.7	234 (53.6)	232–236	70 (14.6)	69–71

a Values are kilocalories/(Weight in kg)^3/4^.

b There were 397 dogs in these studies (were 64 intact females, 26 intact males, 162 neutered males and 145 spayed females).

c There were 527 cats in these studies (61 intact females, 15 intact males, 227 neutered males and 224 spayed females).

The Institutional Animal Care and Use Committee, Hill's Pet Nutrition, Inc., Topeka, KS, USA (Permit numbers CP13 and CP14) after review approved these studies. All cats were immunized against the feline panleukopena virus, feline calicivirus, viral rhinotracheitis and rabies. All dogs used in these studies were immunized against canine distemper, adenovirus, parvovirus, bordetella, and rabies. All dogs and cats were healthy and had no signs of systemic disease. This was concluded on the basis of urinalysis, serum biochemical analysis, complete blood count and physical exams all evaluated on a yearly basis in an annual exam. The dogs were allowed to exercise in groups in indoor runs and housed individually. Cats were also individually housed and had indoor runs where daily interaction with other cats and exercise was allowed. Both cats and dogs were housed with access to natural light and with varied seasonal changes. Both cats and dogs had enrichment activities and opportunities for socialization. Enrichment included interactions and play with toys, other cats or dogs and with animal care workers.

The test means (using test as the experimental unit) of a subgroup of these data were previously used to explore the effect of ingredient source on digestibility (11). Less than 20% of these data (using test as the experimental unit) were part of the data set used to estimate each species energy digestibility equation for the combined dry and wet foods (7). Initial feeding amounts were based on either previously measured energy density with similar formulae, manufactures statements as to energy density, energy density based on Hall et al. (7), or on modified Atwater predictions (3, 4).

The canine and feline foods varied in gross energy, energy digestibility, moisture, protein, fat, ash and crude fiber as reported in Tables 2, 3 for cats and dogs, respectively. The foods represent the wide spectrum of available foods including foods designed to aid in the management of disease, foods designed for specific life stages or conditions, and foods designed to meet the needs of all life stages. The main ingredients generally were animal proteins (meats, meat meals, eggs), grains and high carbohydrate ingredients (rice, corn, wheat, sorghum, potato, quinoa), and plant proteins (corn gluten meal, rice protein concentrate, soybean either as a meal or an isolate) with oils (animal fat, soybean oil, fish oil, flax oil) and vitamins and minerals. This study used the measurements of gross energy, moisture, protein, crude fat, ash, and crude fiber to predict energy density. The measurement of these dietary factors was completed as previously outlined (2) using the methods of the Association of Analytical Communities (AOAC) by a commercial laboratory. Food analytical measurements for energy, moisture, protein, fat, crude fiber and ash were performed as outlined by AAFCO (2). Food composition of the experimental foods was determined by a commercial laboratory (Eurofins Scientific, Inc., Des Moines, IA) using Association of Analytical Communities methods (moisture—AOAC 930.15; protein—AOAC 2001.11; fat—AOAC 954.02; fiber—AOAC 962.09; and ash—AOAC 942.0).

**Table 2:** Characteristics of cat foods tested.

	**Dry cat foods (*****n*** = **506)**					**Wet cat foods (*****n*** = **341)**				
	**Mean**	**Standard deviation**	**95% Confidence interval**	**Median**	**Range**	**Mean**	**Standard deviation**	**95% Confidence interval**	**Median**	**Range**
Moisture (%)	6.50	1.25	6.4–6.6	6.40	2.4–11.1	77.9	2.78	77.7–78.1	78.4	65.8–84.7
Protein (%)	33.3	4.53	33.1–33.5	32.8	22.8–57.8	8.54	1.69	8.45–8.63	8.30	5.0–15.0
Fat (%)	18.0	3.91	17.8–18.2	18.6	3.1–31.7	5.03	1.55	4.95–5.11	5.00	2.0–13.8
Crude fiber (%)	3.01	2.63	2.89–3.13	2.0	0.2–14.9	1.04	0.81	1.00–1.08	0.80	0.1–5.4
Ash (%)	5.45	1.47	5.38–5.52	5.20	3.5–30.1	1.35	0.27	1.34–1.36	1.30	0.6–2.5
Gross energy^a^	5,106	249.8	5,095–5,117	5,142	4,009–5,815	1,245	182.4	1,235–1,254	1,205	844–2,172
Metabolizable energy (ME)^a^	4,199	354.5	4,183–4,215	4,247	2,786–4,871	984	176.9	975–994	969	603–1,840
Within test ME standard deviation^b^	121.8	55.5	119.3–124.3	113.6	0.0–528.6	36.6	21.3	35.4–37.8	32.4	0.8–204.7
Digestible energy (%)	87.2	3.99	87.0–87.4	88.0	74.1–94.0	83.9	5.26	83.6–84.2	85.3	61.4–92.8

a Values (kcals/kg) are from tested ME using the AAFCO protocols.

b Values (kcals/kg) are the within test standard deviation reflecting the variation within each individual test.

**Table 3:** Characteristics of dog foods tested.

	**Dry cat foods (*****n*** = **711)**					**Wet cat foods (*****n*** = **317)**				
	**Mean**	**Standard deviation**	**95% Confidence interval**	**Median**	**Range**	**Mean**	**Standard deviation**	**95% Confidence interval**	**Median**	**Range**
Moisture (%)	8.41	1.06	8.37–8.45	8.40	3.5–12.2	77.0	3.86	76.9–77.1	76.5	63.8–84.4
Protein (%)	22.5	5.41	22.3–22.7	22.3	9.9–54.2	5.46	1.36	5.41–5.51	5.20	1.90–11.3
Fat (%)	14.8	3.91	14.6–15.0	14.4	6.7–35.9	4.10	1.73	4.04–4.16	3.70	0.6–9.8
Crude fiber (%)	3.66	3.37	3.53–3.79	2.40	0.5–21.8	1.17	0.90	1.14–1.20	0.90	0.1–4.6
Ash (%)	5.24	1.13	5.20–5.28	5.10	2.3–14.6	1.28	0.30	1.27–1.29	1.20	0.6–2.5
Gross energy^a^	4,693	247.7	4,684–4,702	4,670	3,987–6,123	1,176	223.3	1,167–1,184	1,159	438–1,990
Metabolizable energy (ME)^a^	3,808	348.5	3,793–3,819	3,851	2,453–4,933	928	225.9	920_936	896	216–1,713
Within test ME standard deviation^b^	76.0	37.1	74.6–77.4	69.4	11.7–331.6	22.1	13.3	21.6–22.6	19.2	1.0–124.9
Digestible energy (%)	86.1	5.28	85.9–86.3	87.6	59.4–95.4	83.1	5.95	82.9–83.9	84.5	57.6–97.8

a Values (kcals/kg) are from tested ME using the AAFCO protocols.

b Values (kcals/kg) are the within test standard deviation reflecting the variation within each individual test.

A group of responses (~30% of total by random allocation) were assigned as a check group which were not used to establish the new prediction equations. The group of 70% of the responses was used to generate new equations based on species and dietary form (dry or wet). The calculations of variation from measured energy density for all prediction equations were completed to evaluate the accuracy of the energy estimates. The previously established energy prediction statements follow. Gross energy and the estimates are expressed as kilocalories per kilogram. Protein, fat, moisture ash, and crude fiber are expressed as grams/100 grams. Fiber and fat are both the result of the AOAC methods described earlier and are often stated as “crude fat” or “crude fiber.”

Canine ME (Kcal/kg) estimates:


$$\begin{array}{l} \left(modified\;Atwater\right)\\ \;=35{\;}^{*}protein+85{\;}^{*}fat+35{\;}^{*}(100-fat-protein\\ \;-moisture-fiber-Ash)\\ \;(Hall)\text{ ​​​​}=575+(0.8166){\;}^{*}\;GE-(20.616){\;}^{*}\;protein+(12.086)\\ \;{\;}^{*}fat-(6.076){\;}^{*}\;moisture-(52.766){\;}^{*}fiber\\ \;(NRC)\\ \;=10{\;}^{*}\;[((91.2-(1.43{\;}^{*}fiber))/100){\;}^{*}(GE/10)-(1.04protein)] \end{array}$$

Feline estimates:


$$\begin{array}{l} \left(modified\;Atwater\right)\\ \;=35{\;}^{*}\;protein+85{\;}^{*}fat+35{\;}^{*}\;(100-fat-protein\\ \;-moisture-fiber-Ash)\\ \;(Hall)\text{ ​​​​}=-541+(0.923){\;}^{*}\;GE-(4.216){\;}^{*}\;protein+(14.686)\\ \;{\;}^{*}fat+4.806{\;}^{*}\;moisture-44.31{\;}^{*}\;fiber\\ \;(NRC)\\ \;=10{\;}^{*}\;[((87.9-(0.88{\;}^{*}\;fiber))/100){\;}^{*}\;(GE/10)-(0.77{\;}^{*}\;protein)] \end{array}$$

The statistical analysis performed used SAS 9.4 (SAS Institute, Cary, NC, USA). The absolute value of the difference between predicted and measured metabolizable energy (ME) was calculated. These differences were then compared among prediction equations by using PROC MIXED in SAS within the dry and wet food categories of each species, using individual pet identification as a random variable. When the differences were not normally distributed, log values of the differences were used for mean separation. A *p* < 0.05 was used as cut off for significance. Regression analysis was used to generate co-factor coefficients for each species and within the wet or dry categories were generated using PROC MIXED in SAS. Besides the actual dietary factors measured the analysis of the possible predictor factors included the square of each of the factors and the complete list of one way interactions. The addition of a new factor to those considered for coefficients was evaluated through PROC STEPWISE in SAS. If the factor addition was significant (*p* < 0.05) and if it improved the r-square of the overall prediction (*p* > 0.005) then it was included. In order to remain in the model the factor also had to be significant in the PROC MIXED analysis for generation of the coefficients. An overall evaluation of the predictions was performed using species type and check status (used to develop the model or held for independent evaluation) as independent variables. Colinearity was evaluated by using the variance inflation factor (VIF) indicator generated by PROC REG in SAS according to the methods previously defined (12). The VIF (which is 1/(1-${\text{R}}_{\text{i}}^{2}$)) where ${\text{R}}_{\text{i}}^{2}$ is the coefficient of determination for the regression of the *i*th independent variable on all other independent variables increases as the colinearity increases which is related to the instability of the estimate of the coefficients. There is no formal determining maximum for VIF although higher values suggest reduced confidence in the coefficients. However, for prediction purposes the coefficients are less important than the final prediction value (13).

## Results

The dietary intakes of the daily metabolizable energy for dogs and cats are reported in Table 1. There is an increased energy use (when scaled to weight in kilograms to the 3/4 power) in dogs as compared to cats. However, both species have similar and significant variation of pet to pet energy use with one standard deviation 20% of the mean for dogs and 21% for cats (when energy is expressed as kcals/weight^3/4^).

The observed values for concentrations of the macronutrients and energy of the foods used are reported in Tables 2, 3. These values were the results of the variation of pet foods tested and were not selected for any specific values or central tendencies. The mean dry matter energy densities for cat food were (4,490 kcal/kg dry and 4,452 kcal canned) and dog food were (4,155 kcal/kg dry and 4,034 canned). The mean energy digestibility of the dog and cat, dry and canned foods, are also shown in Tables 2, 3. The energy in dry foods was more digestible than canned and the gross energy in the cat food was more digestible than that in dogs. By using previously published methodology (14) the maximum total error acceptable for comparable methodologies can be calculated. In short, this method requires an estimate of the maximum allowed imprecision, the maximum allowed inaccuracy, and total error which is calculated from within animal and between animal variation. This analysis relates to the acceptance of the values of the comparative method and to those of the original method. The maximum total errors for energy density calculated from Tables 2, 3 for dogs are 3.9% for dry and 8.1% for wet food. For cat food the maximum total errors are 3.9% for dry and 7.6% for wet food. The measured ME concentrations of dry and wet cat and dog foods and the estimated ME concentrations of the predictive equations are shown in Tables 4, 5, respectively. The new equations based on species and dietary form (dry or wet) are as follows:

**Table 4:** Feline tested and estimated metabolizable energy (Kilocalories/kg).

	**Dry cat foods (*****n*** = **2,115)^π^**				**Wet cat foods (*****n*** = **1,253)^π^**			
	**Mean**	**Standard deviation**	**Median**	**Range**	**Mean**	**Standard deviation**	**Median**	**Range**
Metabolizable energy (ME)^1^	4,199	375.1	4,272	2,687–5,124	996	187.7	973	124–1,921
Modified Atwater prediction^2^	3,875	270.3	3,941	2,880–4,406	952	162.9	922	600–1,786
Modified Atwater variance^3^	347^a^	181.5	339	0–1,182	70^a^	52.8	62	0–476
Hall prediction^4^	4,190	337.9	4,265	3,011–5,062	986	190.9	953	592–1,897
Hall variance	133^c^	117.0	106	0–1,286	44^c^	42.8	34	0–467
NRC prediction^5^	4,095	276.6	4,163	3,082–4,716	1,027	159.5	995	685–1,484
NRC variance	170^b^	123.9	152	0–1,214	53^b^	53.8	38	0–560
New prediction^6^	4,198	333.2	4,283	3,007–4,951	997	178.1	970	620–1,819
New prediction variance	128^c^	114.2	102	0–1309	40^d^	42.1	30	0–495
	**Dry cat foods (*****n*** = **859)**^Ω^				**Wet cat foods (*****n*** = **601)**^Ω^			
Metabolizable energy (ME)^1^	4,198	372.1	4,253	2,836–5,027	970	158.9	963	609–1,422
Modified Atwater prediction^2^	3,883	264.4	3,909	3,160–4,458	928	143.5	905	665–1,286
Modified Atwater variance^3^	347^a^	193.5	340	0–1,247	61^a^	41.0	57	0–207
Hall prediction^4^	4,202	332.6	4,226	3,362–4,906	959	163.3	937	627–1,403
Hall variance	138^c^	145.8	109	0–1,611	38^c^	28.8	34	0–192
NRC prediction^5^	4,104	270.3	4,118	3,422–4,688	1,005	137.0	981	764–1,366
NRC variance	173^b^	143.5	110	0–1,501	52^b^	46.8	39	0–272
New prediction^6^	4,208	326.5	4,224	33,982–4,891	972	153.4	954	643–1,387
New prediction variance	137^c^	143.8	1,101	0–1,648	35^c^	27.5	29	0–169

π These data (group 1, n = 2,115 dry; 1,253 wet) were randomly chosen as the data set to be used to generate the new prediction equations.

Ω These data (group 2, n = 859 dry; 601 wet) were randomly chosen as a check data set independent of the data used to generate the new prediction equations.

1 ME and all numbers in the table are kcals/kg.

2 Modified Atwater energy = 85^*^fat + 35^*^NFE + 35 ^*^ protein.

3 Variance is the absolute value of the difference between the measured ME and the prediction.

4 Hall et al. prediction equation ME defined in the paper.

5 NRC crude fiber prediction equation ME defined in the paper.

6 New prediction equations which were generated from the 2,115 cat data set (dry foods) and 1253 cat data set (wet foods).

ME defined in the paper.

a, b, c, d Means with different superscripts in the same column and group are different p < 0.01.

**Table 5:** Canine tested and estimated metabolizable energy (kilocalories/kg).

	**Dry dog foods (*****n*** = **2,843)^π^**				**Wet dog foods (*****n*** = **1,326)^π^**			

	**Mean**	**Standard deviation**	**Median**	**Range**	**Mean**	**Standard deviation**	**Median**	**Range**
Metabolizable energy (ME)^1^	3,820	345.1	3,861	1,951–4,819	926	230.6	896	112–1,779
Modified Atwater prediction^2^	3,638	240.7	3,658	2,750–4,440	920	197.0	913	572–1,662
Modified Atwater variance^3^	216^a^	141.6	202	0–1,485	59^b^	85.7	41	0–1,043
Hall prediction^4^	3,889	302.5	3,924	2,723–4,679	941	226.0	908	442–1,732
Hall variance	114^b^	108.3	90	0–1,671	35^c^	46.0	25	0–480
NRC prediction^5^	3,814	296.1	3,872	2,561–4,625	994	203.6	966	355–1,712
NRC variance	106^c^	102.5	84	0–1,701	73^a^	58.4	58	0–525
New Prediction^6^	3,849	316.1	3,897	2,637–4,649	925	224.7	893	231–1,706
New prediction variance	82^d^	82.6	66	0–1,172	22^d^	19.5	17	0–156
	**Dry dog foods (*****n*** = **1,416)**^Ω^				**Wet dog foods (*****n*** = **570)**^Ω^			
Metabolizable energy (ME)^1^	3,778	371.6	3,835	2,472–4,986	926	215.5	875	475–1,578
Modified Atwater prediction^3^	3,619	291.2	3,642	2,666–4,721	927	191.8	900	603–1,490
Modified Atwater variance^4^	205^a^	143.5	189	0–1,176	51^b^	40.1	44	0–245
Hall prediction^5^	3,855	354.0	3,900	2,634–4,877	943	219.6	892	546–1,689
Hall variance	124^b^	106.9	95	0–728	33^c^	30.6	26	0–265
NRC prediction^6^	3,778	357.7	3,838	2,447–4,693	999	187.9	973	622–1,689
NRC variance	109^c^	101.2	83	0–916	77^a^	58.6	63	0–319
New prediction^7^	3,814	366.9	3,863	2,499–4,731	926	214.2	879	499–1,672
New prediction variance	89^d^	81.8	75	0–603	31^c^	29.0	25	0–268

π These data (group 1, *n* = 2843 dry; 1326 wet) were randomly chosen as the data set to be used to generate the new prediction equations.

^ω^These data (group 2, *n* = 1416 dry; 570 wet) were randomly chosen as a check data set independent of the data used to generate the new prediction equations.

1 ME and all numbers in the table are kcals/kg.

2 The average of the absolute value of the individual pets estimate as compared to the 6 pet mean.

3 Modified Atwater energy = 85^*^fat + 35^*^NFE + 35^*^protein.

4 Variance is the absolute value of the difference between the measured ME and the prediction.

5 Hall et al. prediction equation ME defined in the paper.

6 NRC crude fiber prediction equation ME defined in the paper.

7 New prediction equations which were generated from the 2,843 dog data set and 1,346 dog data set (wet foods).

a, b, c, d Means with different superscripts in the same column and group are different p < 0.01.

Canine ME (Kcal/kg) estimates:


$$\begin{array}{l} \left(\;New\;Dry)=48.7+(0.934){\;}^{*}\;GE-(7.21){\;}^{*}\;moisture-(50.19\right)\\ \;{\;}^{*}\;protein+(64.9){\;}^{*}\;fat-(41.2){\;}^{*}\;fiber-(13.4){\;}^{*}\;ash\\ \;+(0.00736){\;}^{*}\;GE{\;}^{*}\;protein-(0.0126){\;}^{*}\;GE{\;}^{*}\;fat\\ \;-(3.323){\;}^{*}\;fiber{\;}^{*}\;ash\\ \left(New\;Wet)=-\;307.7+(1.072){\;}^{*}\;GE+(1.29){\;}^{*}\;moisture+(9.85\right)\\ \;{\;}^{*}\;protein-(4.90){\;}^{*}\;fat-(46.1){\;}^{*}\;fiber+(10.49){\;}^{*}\;ash\\ \;-(0.0182){\;}^{*}\;GE{\;}^{*}\;protein+(8.134){\;}^{*}\;fat{\;}^{*}\;fiber\\ \;-(25.06){\;}^{*}\;fiber{\;}^{*}\;ash \end{array}$$

Feline estimates:


$$\begin{array}{l} \;(New\;Dry)=-505.5+(0.9233){\;}^{*}\;GE-(0.432){\;}^{*}\;moisture\\ \;-(0.3719){\;}^{*}\;protein+(13.1){\;}^{*}\;fat-(12.65){\;}^{*}\;fiber\\ \;+(14.57){\;}^{*}\;ash-(0.402){\;}^{*\;}protein{\;}^{*}\;fiber\\ \;-(0.7567){\;}^{*}\;protein{\;}^{*}\;ash-(1.794){\;}^{*}\;fiber{\;}^{*}\;fat\\ \left(New\;Wet)\;=-\;419+(0.9462){\;}^{*}\;GE+(4.26){\;}^{*}\;moisture-(10.79\right)\\ \;{\;}^{*}\;protein+(4.59){\;}^{*}\;fat-(48.69){\;}^{*}\;fiber+(10.79){\;}^{*}\;ash \end{array}$$

These previously published and new equations were evaluated for comparative benefit in predicting ME by comparing the means of the absolute values of the difference between the predicted and measured ME. The feline dry and wet food energy density estimates were most accurate with the new equations followed by the Hall equation. Both of these estimates were better than the NRC estimates which in turn were superior to the modified Atwater estimates (Table 4).

The dog food energy estimates for dry food are best using the newly derived equation, which was followed by the NRC equation, followed by the Hall equation while the modified Atwater equation had the worst estimates of energy density for dry dog food. For the canine wet foods the best estimate of ME was through using the newly derived equation, followed by the Hall equation which was followed by the modified Atwater equation, while the NRC prediction had the most variance from the measured ME (Table 5).

The prediction values listed in Tables 4, 5 all had significant slopes (*p* < 0.01) when regressed to measured energy digestibility. There was an overestimation of ME when energy digestibility was low and an underestimation when energy digestibility was high in all equations. The intercepts of energy digestibility for the feline dry and wet forms for modified Atwater prediction were 75.4 and 78.3%; Hall prediction intercepts were 86.8 and 81.0%; NRC prediction intercepts were 83.2 and 87.4%; The new feline prediction intercepts were 87.1 and 85.9%; dry and canned, respectively. In the dog the intercepts for modified Atwater predictions were 78.2 and 82.6%; Hall prediction intercepts were 92.1 and 90.4%; NRC prediction intercepts were 85.7 and 91.9%; The new canine prediction intercepts were 89.5 and 82.6%; dry and canned respectively. The scatter plots of each estimate of energy density as plotted against measured ME are shown in Supplementary Figures 1–16. Because there was not always a difference between the variance of the prediction equations in all of the above groupings an overall evaluation was conducted. This showed that in the data from which the new predictions were calculated, as well as in an independent analysis with the data not included in the development of the equations, the variations were modified Atwater>Hall>NRC>New with each prediction method being different from all others.

## Discussion

These studies show that the AAFCO digestibility technique has a robust ability to measure dietary metabolizable energy over a broad spectrum of energy concentrations in dog and cat foods. This conclusion is based on the standard deviation (expressed as coefficient of variance) as an estimate of the within study variation which was always below 4%. This shows low biological variability in the test which leads to the reasonable conclusion that the test is repeatable with a robust ability across quite variable energy densities. As the analytical variance is equally low these CV% reflect a repeatable and valuable assay. If there were no constraints in resources, the biological measurement of ME is clearly an excellent and superior technique allowing both optimum nutrient to energy ratios and limiting the environmental overload resulting from imbalanced nutrition. The equations using the food analysis to evaluate measured metabolizable energy in this study take different approaches to estimating energy density. The modified Atwater equation uses coefficients that estimate the energy in carbohydrate (as represented by the amount of food which is not moisture, protein, fat, ash or crude fiber), protein and fat. This has a great potential for bias (and subsequent error) as the digestibility for each macronutrient increases above what was used to generate the coefficients. The underestimated ME estimated by using the modified Atwater coefficients in highly digestible pet food has been repeatedly shown (7, 15–17). Intuitively, using the extra information associated with gross energy is a significant enhancement as compared to the modified Atwater factors. However, all equations are limited by the data set from which they are derived and may be enhanced by increasing information describing the food whose energy density is being estimated. In this evaluation the equations which used gross energy were all superior to the modified Atwater estimates which do not use it. All estimates of ME have at their base an estimate of the foods energy digestibility and all equations have error associated with the prediction vs. actual energy digestibility. When the relationship between the delta of the predictions from the measured ME (the prediction estimate subtracted from measured ME) was regressed against measured energy digestibility the modified Atwater prediction underestimated energy concentration in dry food when energy digestibility was above 78.2 (canine) and 75.4% (feline). As the average pet food in this analysis exceeded these energy densities this relationship partially explains the underestimated energy density observed using the modified Atwater prediction. The NRC equations start with the measured gross energy and then estimate the loss of energy associated with changes in dietary fiber and protein. These prediction equations are also influenced by energy digestibility. In the case of these equations the energy digestibility intercepts for dry food (85.7% canine, 83.2% feline) were closer to the actual energy digestibility in these data. The Hall equations were also influenced by energy digestibility with the intercept (92.1 canine and 86.8% feline) showing a computational bias toward a more correct answer with higher digestible energy foods as compared to either the NRC equations or the modified Atwater equation. Finally, the newly derived equations have intercepts in the dog (86.5% and 83.0% dry and wet, respectively) and feline (87.2% and 84.2%, dry and wet, respectively). These also show the bias toward better predictions for foods with higher energy digestibility. This discussion on the relationship of the prediction to actual energy digestibility highlights a weakness of all prediction equations which is that they are tuned to the data set from which they are derived. It is interesting in this case that the data set used to derive the equations and the group used to check the derived equations resulted in similar and precise average deltas from the measured ME (~3% of ME). This suggests they are robust equations. However, it also could suggest that both groups had similar foods with similar ingredients and energy digestibility, thus providing a good match for the derived equations.

The errors associated with both imprecision and inaccuracy of prediction equations are summed in the absolute value of the difference between the estimated and the measured metabolizable energy. This analysis allows the variance to be analyzed on an individual pet basis which increases the variation estimate from what is estimated from the prediction equation and the means of the group in which the food was tested. The individual pet data also allows a calculation of a maximum acceptable difference for comparison of methods. The maximum allowable total error calculation has previously reported and is a function of both within test and between test variations (14). When looking at the data from this perspective, the equations generated from the modified Atwater equations exceed the maximum total error for both canine and feline dry products. All other equations have equal or less than the calculated total error maximums. However, because there is still significant error associated with the prediction equations (Tables 4, 5) none of them achieve the goal of making the measured metabolizable energy a redundant measure. It remains then to evaluate these equations with respect to their specific uses.

Previous research (15) has reported that the overall best equations for estimating energy density were the NRC equations that use total dietary fiber as compared to the equations that used crude fiber. This current study cannot evaluate the improvements that may be associated with using total dietary fiber as it was not measured in enough of these studies to allow for a meaningful comparison. Unfortunately, because the analysis done previously (7, 15, 16) was completed using the digestibility study as an experimental unit (as compared to the individual pet) it is not directly possible from those evaluations to establish an acceptability of error using the variation associated with the within test pet to pet variation in the metabolizable energy tested evaluation. Also, the knowledge of the variation of the individual pet energy need (as well as the individual's ability to digest energy) informs the evaluation of metabolizable energy and this was not previously reported in these studies.

When using estimates of energy density for feeding guides, the variation between each pet associated with energy use is of significantly greater importance than the variation from any of these estimates of energy density. For example in this analysis an average dog of 11.5 kg consumed 747 kcals/day. A standard deviation change in energy use results in a change of 198.7 kcals per day. If the pet food contains the energy density of 3,806 kcals/kg the grams change difference based on this energy use is 52 grams. The mean variance of the worst canine estimate (modified Atwater dry food) has a mean error of 216 and a standard deviation of 141.6 kcals/kg. This example then estimates that a standard deviation of increased error added to the average of the worst estimated ME changes the energy density by 358 kcals/kg food. This calculates to a change of food intake of <20 grams/day for the 11.5 kg dog. This illustration shows that the variation associated with the energy estimates of the pet food is significantly smaller than the variation associated with energy demand of the pet. The conclusion therefore is that the measurement or the estimate of the energy use for each individual pet must be completed by establishing the food required for weight maintenance for each pet. So, the feeding amount is reasonably established by a starting point using any of these methods to establish energy density and then refined for each pet. This is done by feeding, weighing, changing food offering, and weighing again until energy consumed is equal to energy used. It has been reported that most dog owners in Canada used this method to establish the correct amount of food to be offered daily (8).

However, the value of knowing the energy density is established nutritionally because nutrients are scaled to energy density. This is done to avoid both underfeeding and overfeeding nutrients. The prediction variance can lead to both under formulating or over formulating specific nutrients. For example if using the modified Atwater prediction for feline dry foods the food would be calculated to contain ~ 93% of the actual ME density. Therefore, on a calorie basis the food would have a >7% under formulation of protein. If using the NRC equation for wet dog food the formulation would have a calculated energy density more than 3% above the actual energy density. In this case, on a calorie basis the food would have a 3% over formulation of protein. There is a significant improvement in energy density prediction using the formulas which were created not only for each species but also for the dry and wet forms. In this case the average variation from measured metabolizable energy was 3.2%. It's not only the average change between the predictions and the measured energy density that has value. The reduced variation (of the delta between observed and measured ME) observed in the new species and form specific equations impart a value in reducing both the over and under formulation of nutrients. Therefore, using these new equations could enhance optimal nutrition through the improved nutrition to energy ratios. For example, again using protein for a pet that is on the lower end of energy consumed to maintain weight, another 7% decline in protein consumed (because of the error associated with predicting energy density) could come close or even surpass the normal nutritional over formulation used to assure delivery of the minimum protein required. Similarly, nutritional overfeeding could be a problem for a pet that is consuming a high amount of calories on a daily basis where excess nutrients would be consumed. For example the increased fecal ammonia and branched chain fatty acids of over feeding highly available phosphorus has been reported (17). Similarly, overfeeding protein has been shown to have increased fecal ammonia and branched chain fatty acids (18). These examples show there can be negative effects from overfeeding of nutrients and this is exacerbated by incorrectly estimating energy density.

The two most significant issues influencing the sustainability and environmental impact of pet food are nutrient composition and ingredient selection (19). Nutrient composition is directly the result of expected energy density. Therefore, from an environmental standpoint incorrectly estimating energy density is of significant concern. Overestimating energy density can be one of the causes of an increased supplementation of nutrients of concern such as phosphorus, protein and metals (e.g., iron and copper). Although each species has a different carbon footprint for protein output, and the use of proteins not competing for human consumption for protein supplied in pet food is significant, it is still correct that protein is the most expensive nutrient from both an ecological and economic perspective. Using protein as an example–the best delivery of appropriate protein is done through measuring ME. However, the improvement of energy density estimate through using the new equations would reduce protein used and subsequent protein waste as compared to the other equations evaluated in this study. Achieving a nutritional optimum for protein supplementation is not an easy task. It involves not simply the protein to energy ratio but also knowledge of digestibility and the amino acid profile of the protein in the food (protein quality). The combination of the nutritional need and the protein quality of the food allow the calculation of the optimum amount delivered to the pet. This optimum is influenced by food intake (as we've noted above) and also genetic variation, pet health, life stage and lifestyle. Nevertheless, a correct understanding of the energy density of the food improves the protein nutrition delivered independently of these other factors. This applies to phosphorus, iron and copper as well. In a global environmental impact of pet food evaluation (20) it was concluded that pet food (which was estimated to be produced at 2.66 x 10^10^ kilograms per year) had an environmental impact associated of 0.8–1.2% of global agricultural land use. It is apparent that a better calculation of metabolizable energy density allowing an improvement in the protein to energy ratio delivered in the pet food will be a part of the sustainability of pet foods in the future. Currently, the dry matter basis concentration of protein in pet food is 31.4% for dry and 40.8% for wet (21). Although the mix of dry and wet is not known a conservative estimate of protein use in pet food exceeds 1 x 10^10^ kilograms. Clearly, even a 1 or 2% change as a result of better estimates of energy density is a significant environmental benefit. Regarding minerals, an improvement in formulation based on an improved energy density calculation is perhaps less impactful than that of protein. However as seen in swine food (22) an improvement in the nutritional supplement of excess zinc and copper is an environmental benefit.

This study is limited because it was a retrospective analysis. This limitation restricted the opportunity to use coefficients of interest as food analysis was completed for other purposes (and misses some factors which could be quite impactful for estimating energy density). Although there is a reasonable spectrum of variable foods represented another limitation is that the generated equations have the central tendency of foods used. In this data set the foods have a higher energy digestibility than many foods in the market place. This limits the accuracy of the estimated energy density, especially for lower digestible foods. Future research to evaluate more dietary variables and the effect of specific ingredients could further improve prediction equations.

## Conclusion

The new equations, based on both species and product form could be of value in providing optimum nutrition while simultaneously reducing the unneeded over formulation and enhance the environmental footprint of pet food.

## Data availability statement

The raw data supporting the conclusions of this article will be made available by the authors, without undue reservation.

## Ethics statement

The animal study was reviewed and approved by Hill's Pet Nutrition Institutional Animal Care and Use Committee.

## Author contributions

DJ designed and oversaw the experiment. MJ and DJ analyzed, summarized the data, and wrote and edited the manuscript. Both authors have read and agreed to the published version of the manuscript.
