# Supplementary figures and images for: Predictive equations for dietary energy are improved when independently developed for dry and wet food which could benefit both the pet and the environment
Source: Front Vet Sci. 2023 Feb 21;10:1104695. doi: 10.3389/fvets.2023.1104695 (PMC9990867; doi:10.3389/fvets.2023.1104695)

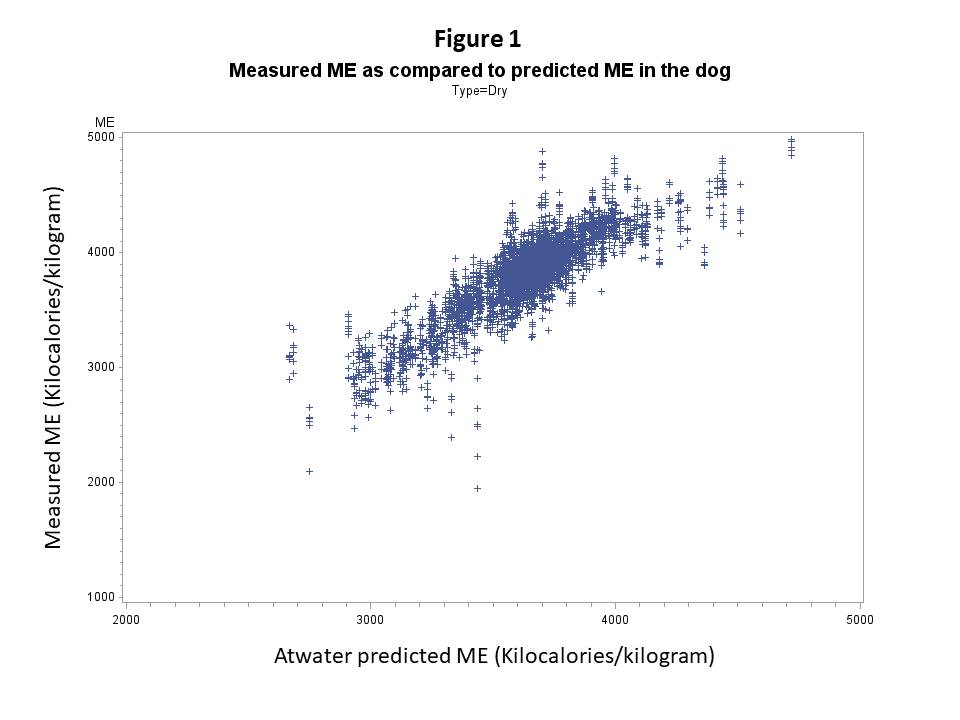

Supplement: Supplementary file 1 [file Data_Sheet_1.zip › Figure 1_v1.jpg]

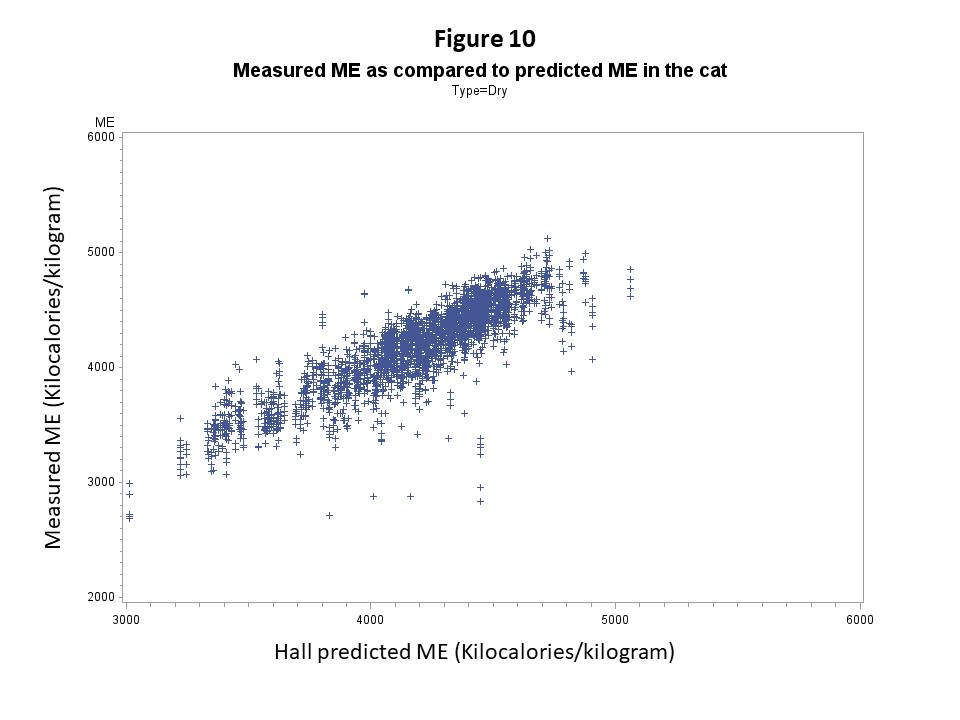

Supplement: Supplementary file 1 [file Data_Sheet_1.zip › Figure 10_v1.jpg]

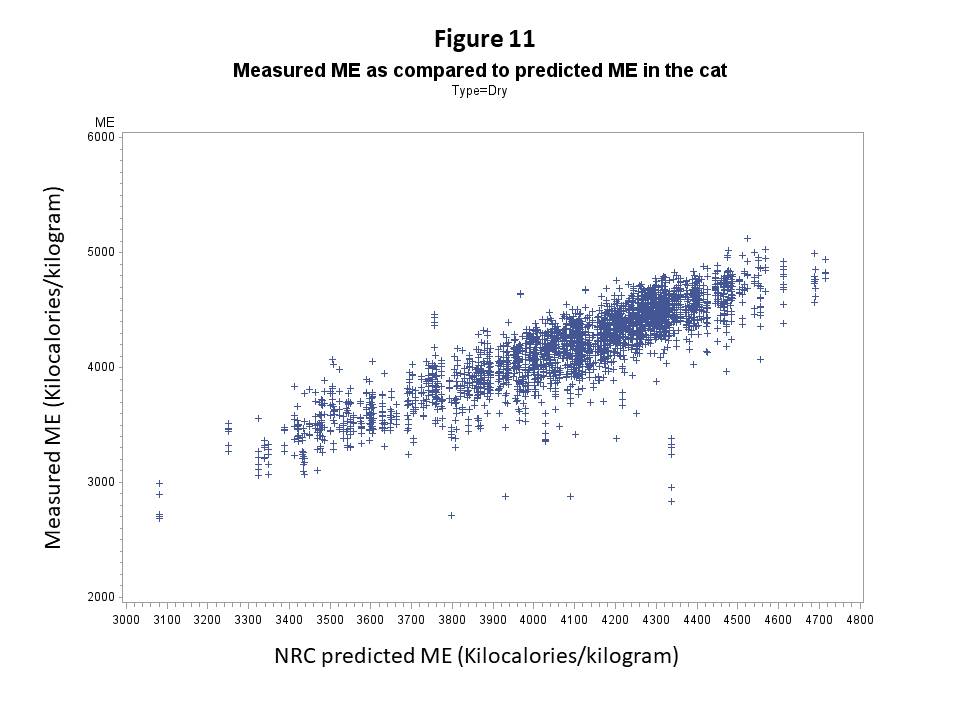

Supplement: Supplementary file 1 [file Data_Sheet_1.zip › Figure 11_v1.jpg]

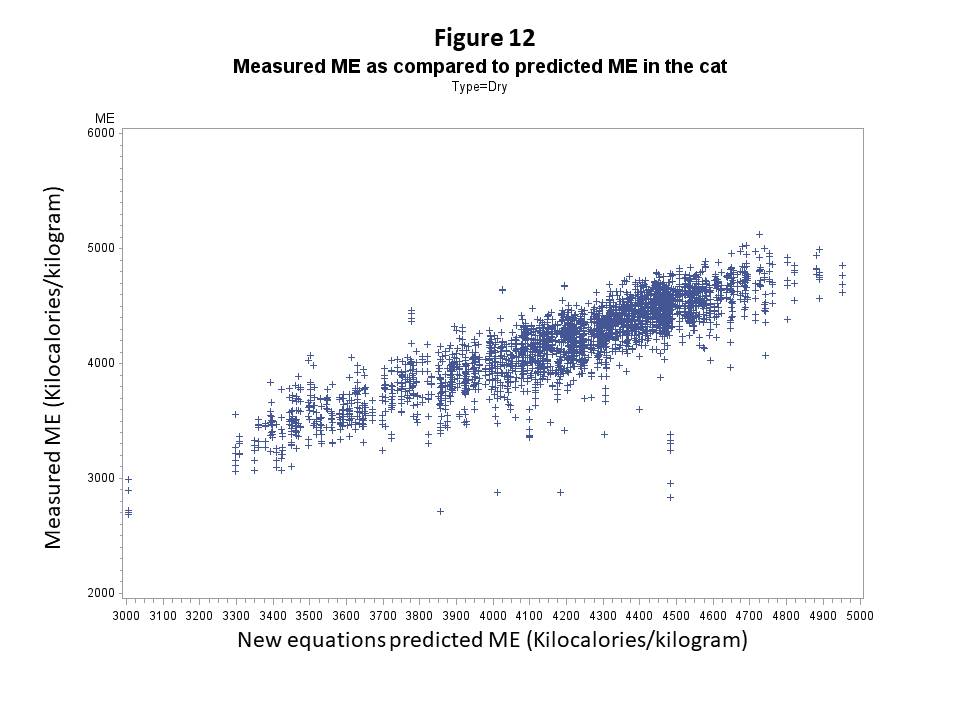

Supplement: Supplementary file 1 [file Data_Sheet_1.zip › Figure 12_v1.jpg]

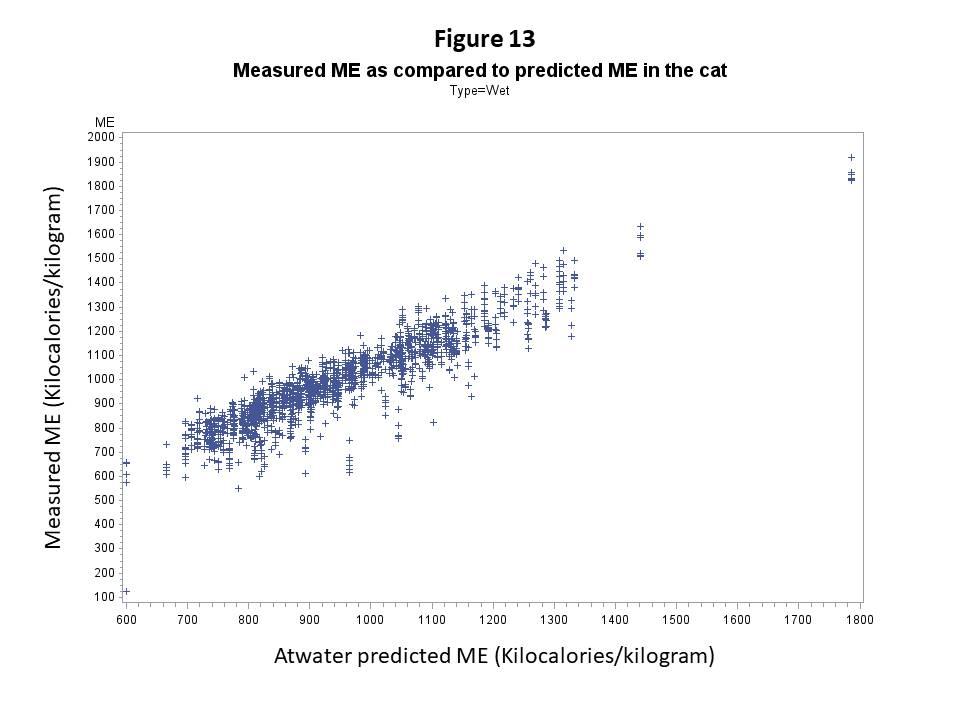

Supplement: Supplementary file 1 [file Data_Sheet_1.zip › Figure 13_v1.jpg]

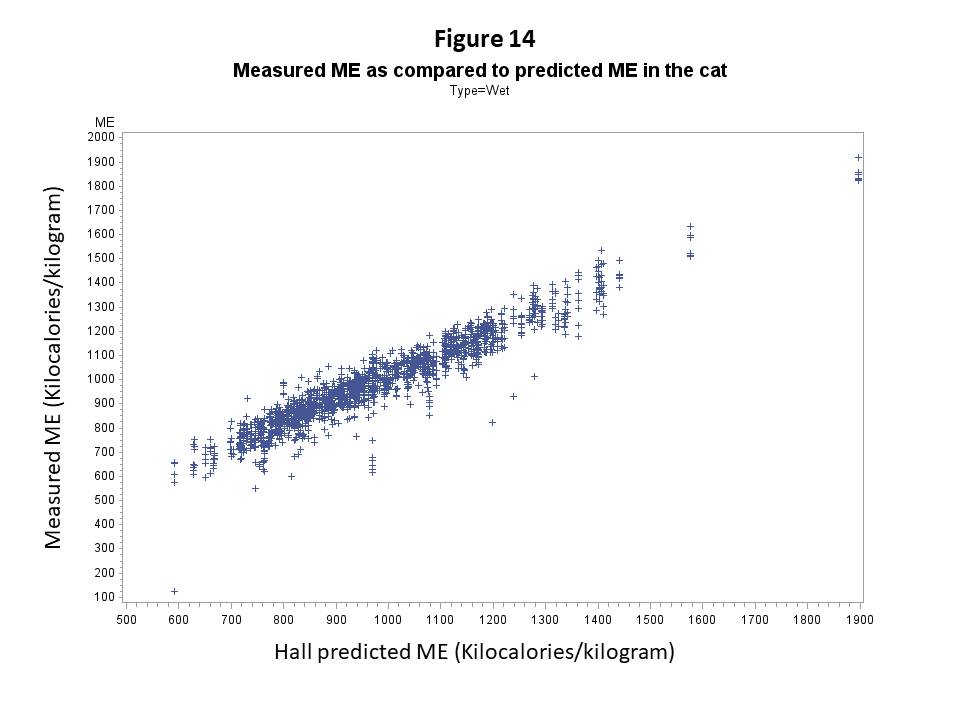

Supplement: Supplementary file 1 [file Data_Sheet_1.zip › Figure 14_v1.jpg]

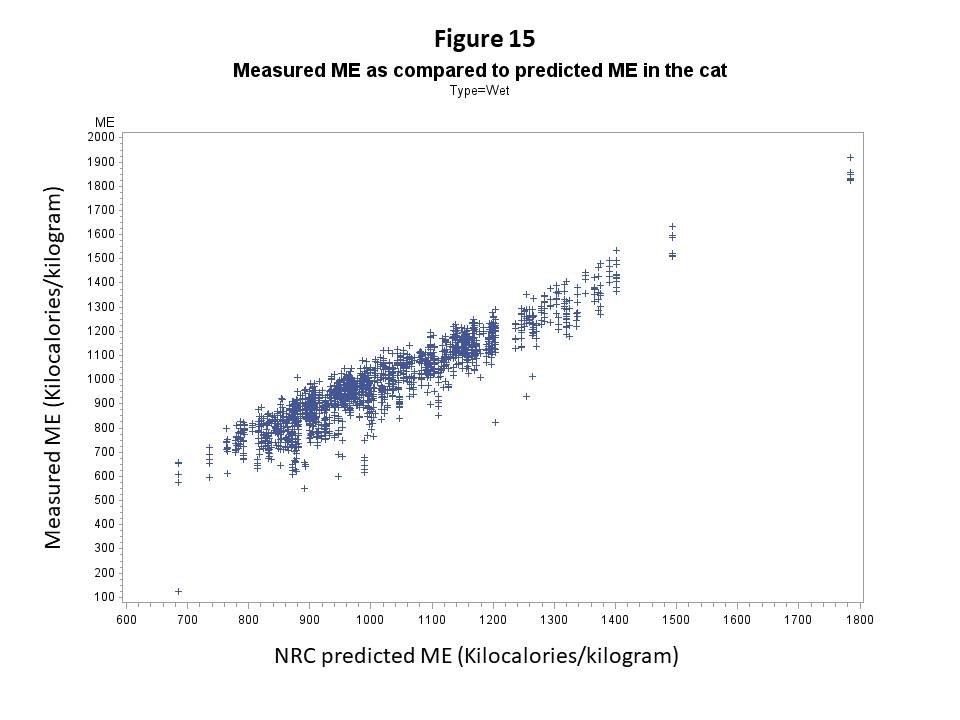

Supplement: Supplementary file 1 [file Data_Sheet_1.zip › Figure 15_v1.jpg]

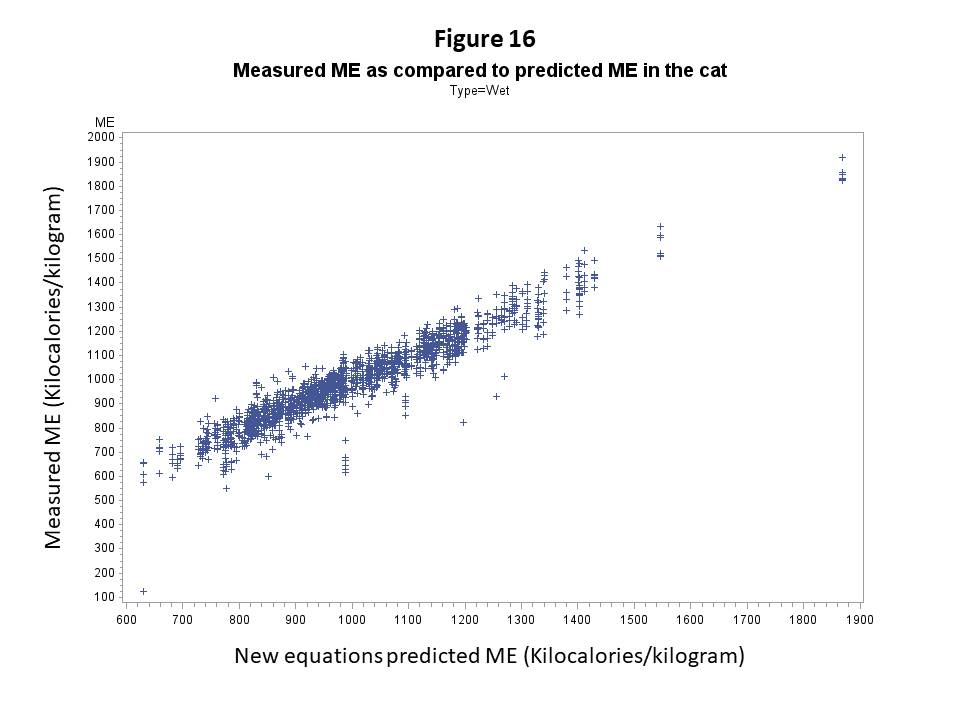

Supplement: Supplementary file 1 [file Data_Sheet_1.zip › Figure 16_v1.jpg]

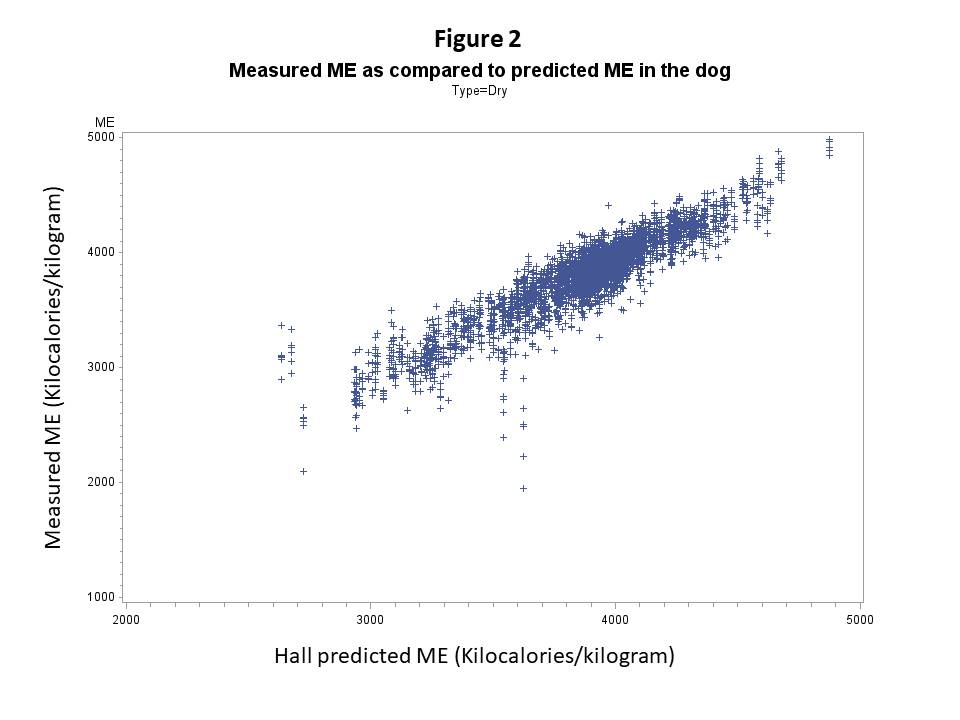

Supplement: Supplementary file 1 [file Data_Sheet_1.zip › Figure 2_v1.jpg]

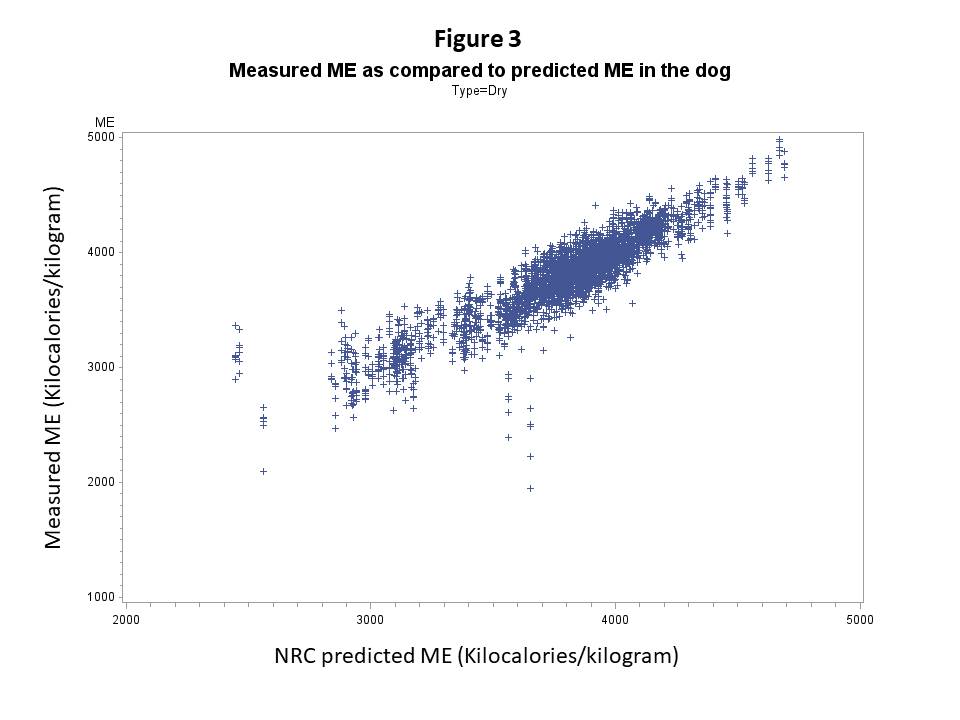

Supplement: Supplementary file 1 [file Data_Sheet_1.zip › Figure 3_v1.jpg]

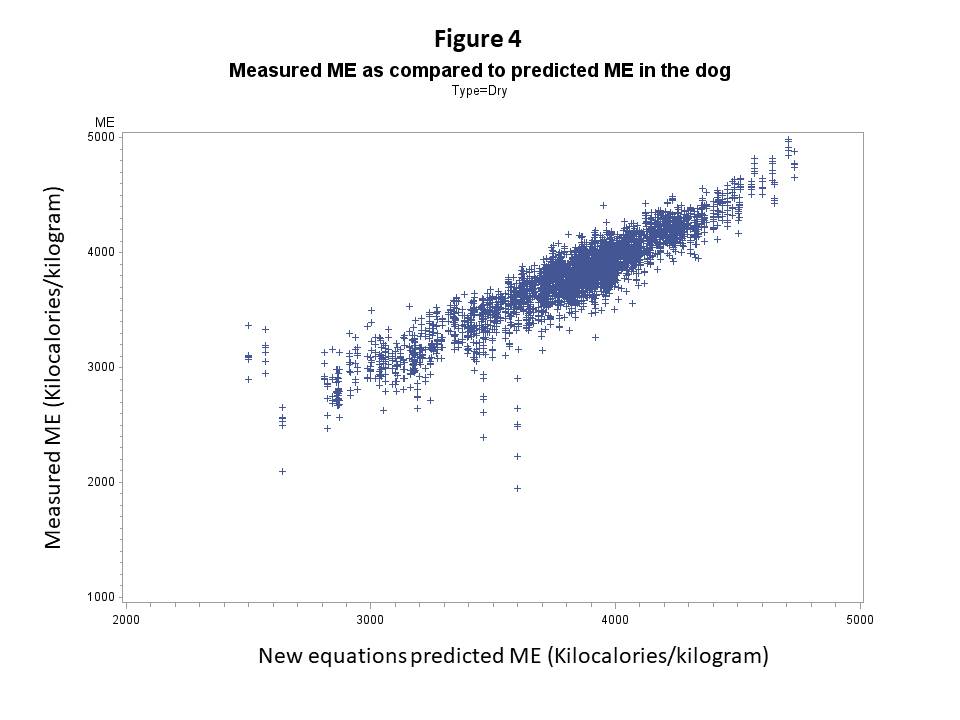

Supplement: Supplementary file 1 [file Data_Sheet_1.zip › Figure 4_v1.jpg]

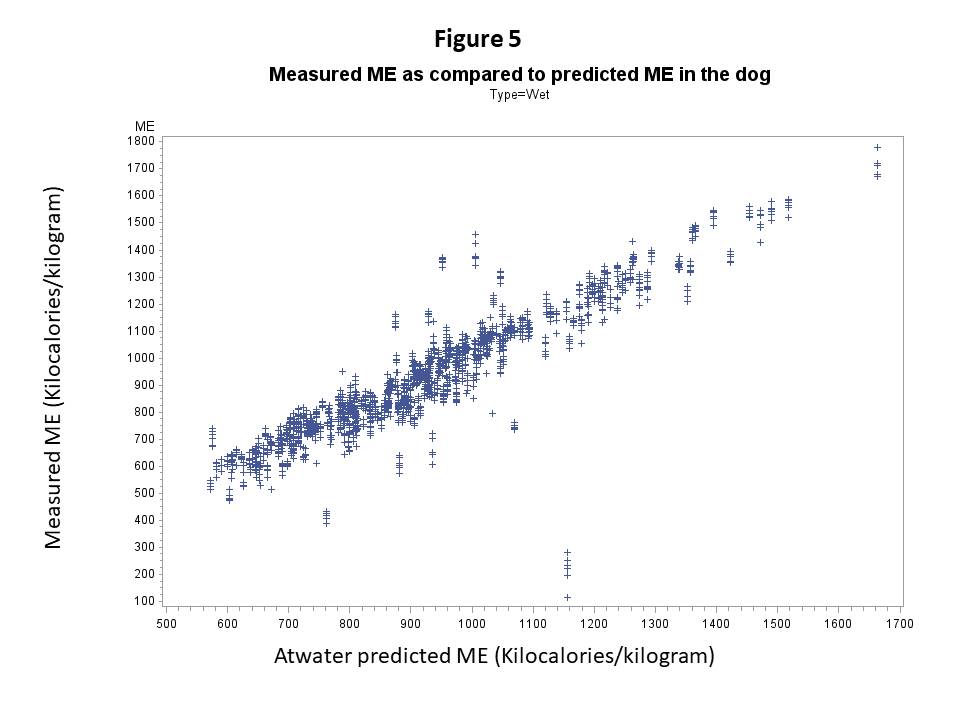

Supplement: Supplementary file 1 [file Data_Sheet_1.zip › Figure 5_v1.jpg]

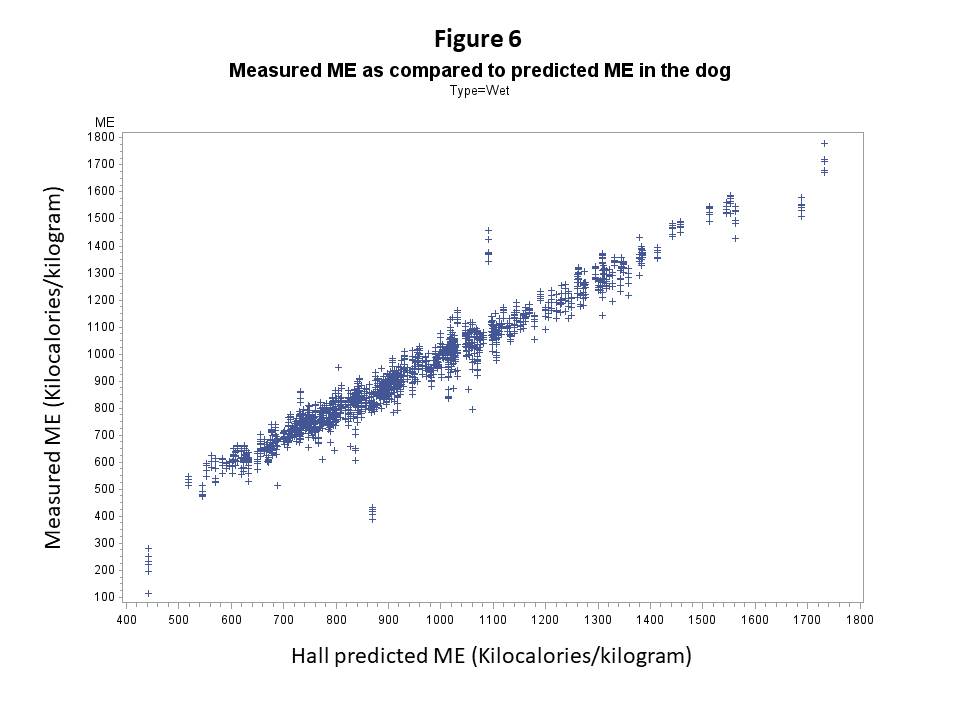

Supplement: Supplementary file 1 [file Data_Sheet_1.zip › Figure 6_v1.jpg]

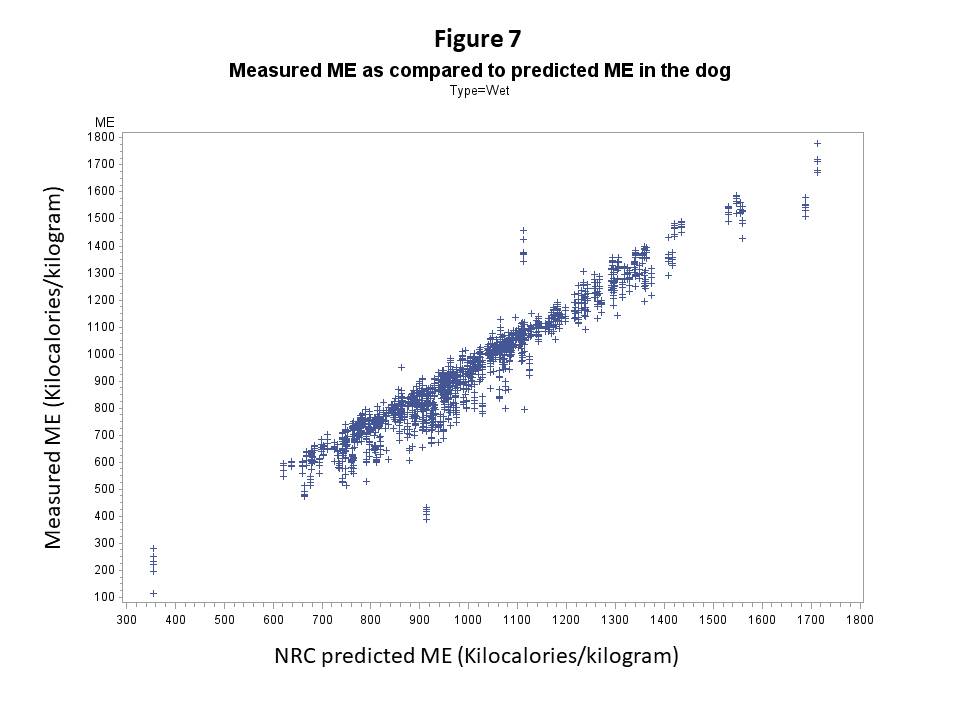

Supplement: Supplementary file 1 [file Data_Sheet_1.zip › Figure 7_v1.jpg]

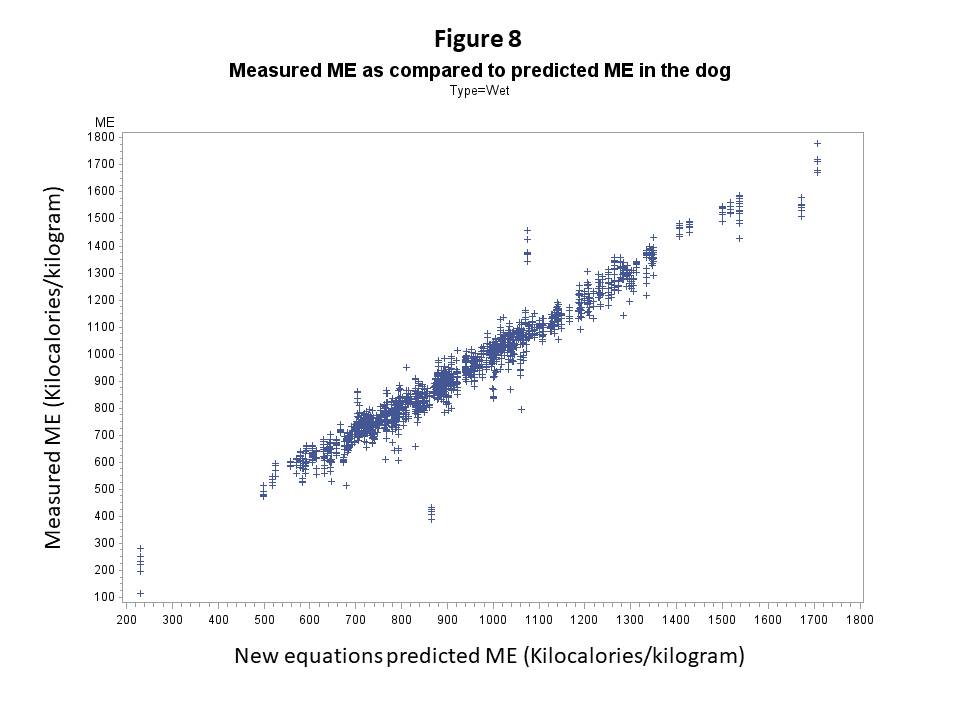

Supplement: Supplementary file 1 [file Data_Sheet_1.zip › Figure 8_v1.jpg]

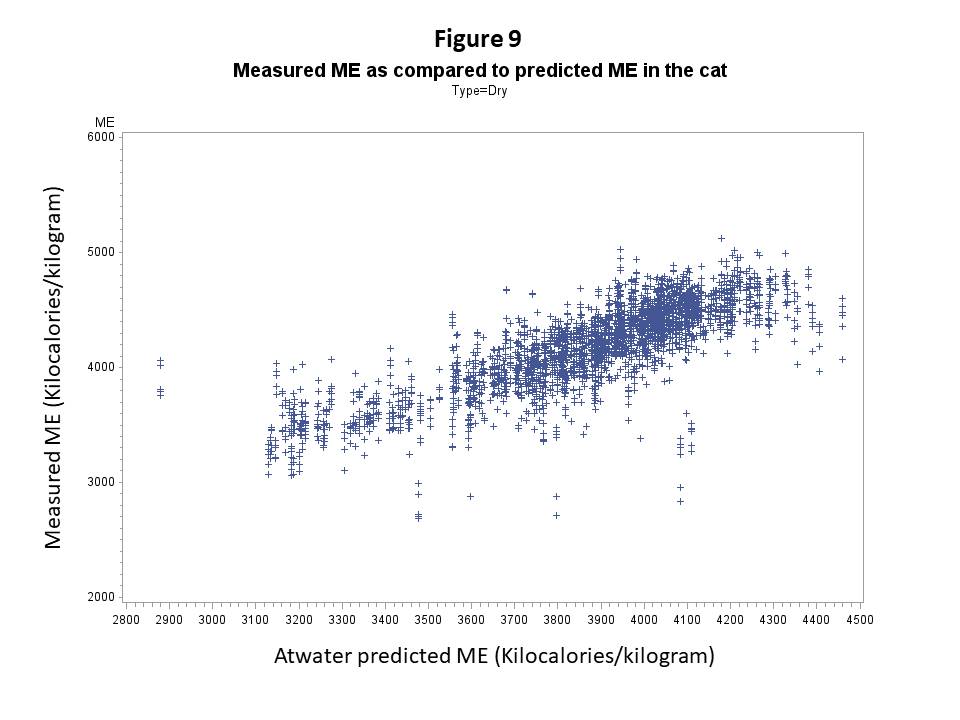

Supplement: Supplementary file 1 [file Data_Sheet_1.zip › Figure 9_v1.jpg]
